# Analysis of membrane fouling by Brunauer-Emmett-Teller nitrogen adsorption/desorption technique

**DOI:** 10.1038/s41598-020-59994-1

**Published:** 2020-02-25

**Authors:** Tiina Virtanen, Gregor Rudolph, Anastasiia Lopatina, Basel Al-Rudainy, Herje Schagerlöf, Liisa Puro, Mari Kallioinen, Frank Lipnizki

**Affiliations:** 1 LUT University, Department of Separation Science, P.O. Box 20, FI-53851 Lappeenranta, Finland; 2 Lund University, Department of Chemical Engineering, P.O. Box 124, SE-221 00 Lund, Sweden

**Keywords:** Chemistry, Engineering, Materials science

## Abstract

Membrane fouling is the major factor limiting the wider applicability of the membrane-based technologies in water treatment and in separation and purification processes of biorefineries, pulp and paper industry, food industry and other sectors. Endeavors to prevent and minimize fouling requires a deep understanding on the fouling mechanisms and their relative effects. In this study, Brunauer-Emmett-Teller (BET) nitrogen adsorption/desorption technique was applied to get an insight into pore-level membrane fouling phenomena occurring in ultrafiltration of wood-based streams. The fouling of commercial polysulfone and polyethersulfone membranes by black liquor, thermomechanical pulping process water and pressurized hot-water extract was investigated with BET analysis, infrared spectroscopy, contact angle analysis and pure water permeability measurements. Particular emphasis was paid to the applicability of BET for membrane fouling characterization. The formation of a fouling layer was detected as an increase in cumulative pore volumes and pore areas in the meso-pores region. Pore blocking was seen as disappearance of meso-pores and micro-pores. The results indicate that the presented approach of using BET analysis combined with IR spectroscopy can provide complementary information revealing both the structure of fouling layer and the chemical nature of foulants.

## Introduction

Wood origninated process and wastewaters contain valuable and underutilized compounds such as hemicelluloses, lignin, phenols and various monomeric compounds. Recovery of these compounds requires efficient separation technologies, and ultrafiltration (UF) has proven to be a very promising technique for fractionation and purification of these compounds from lignocellulosic streams^1^. Unfortunately, the commercial polyethersulfone (PES) and polysulfone (PSU) membranes available for UF processes are rather hydrophobic and thus susceptible to membrane fouling caused by ligneous compounds and wood extractives^2^. Membrane fouling refers to the accumulation of undesirable material on the surface and into the pores of the membrane and is usually accompanied by a loss of membrane performance in terms of flux and retention properties^3^. Due to the complexity and inherent seasonal compositional variation of forest-based streams, a variety of different foulants and fouling mechanisms can be involved in the membrane filtration processes^4^. To enable effective membrane separation of lignocellulosic streams with ultrafiltration, deeper understanding on membrane fouling mechanisms is required. Thus, novel techniques are needed for exploration of the fouling mechanisms and for gaining further knowledge on the chemical compositions and structures of the fouling layer formed in different processes.

In general, the formation of a fouling layer depends on the membrane–foulant and foulant–foulant interactions. Membrane–foulant interactions have a significant effect on the adsorption of foulants on the surface and on the pore walls of the membrane^5^. If the interactions between the membrane material and foulants are favorable, adsorptive fouling can take place in a very short time^6,7^. Characteristics of the adsorptive fouling layer control the subsequent evolution of the fouling because foulant–foulant interactions control the further deposition and aggregation of foulants^5,8,9^.

Size and shape of foulant molecules, along with foulant–foulant interactions determine the structure of the fouling layer. When the size of foulants is smaller than the pore size of the membrane, foulants can enter the pores, adsorb on the pore walls and thus shrink the pore diameter or block the pores. When the size of the foulants is considerably larger than the pore size, foulants cannot enter the pores and the pore size of the selective layer of the membrane remains unchanged^7,8^. Accumulation of foulants on the surface of the membrane can be followed by formation of a gel or cake layer. If the foulants are considerably larger than the pores, the voids between the foulants will as well be larger than the pores of the membrane. The porosity of the fouling layer has been shown to increase with increasing foulant size, and the pore size distribution of voids in the fouling layer is narrower if the foulants are monodispersed and form similar intermolecular openings^10^.

Membrane fouling is typically characterized by measuring the flux decline through membrane or increase in transmembrane pressure. However, verifying adsorptive fouling with such measurements can sometimes be extremely challenging because the effect of the fouling on the membrane flux can be negligible even though the membrane has got fouled. In addition, the conventional measurements cannot distinguish whether changes in membrane performance are caused by adsorption, pore blocking or formation of a cake or gel layer, that all cumulatively affect the flux or pressure^6^. Discrimination between the relative contributions of the different fouling mechanisms is essential in the study of membrane filtration processes, as factors that initiate and enhance membrane fouling can be identified. Due to this, complementary methods are required to get a deeper insight into fouling.

In this study, Brunauer-Emmett-Teller (BET) nitrogen adsorption/desorption technique is applied for organic membrane fouling characterization. BET is a technique that is generally used to determine porosity and surface area of microporous and mesoporous materials. Porosity and surface area are the key parameters in membrane studies as they indicate the structural properties of the membrane and may even reveal the sites for possible foulant accumulation. Amount of the pores, their size distribution, and the most importantly their continuity define also the permeability of the membrane (together with the hydrophilicity of the membrane material). It should be noted that BET method measures all the pores available for nitrogen adsorption and thus it is possible that a sample has a high porosity despite a low permeability due to the high amount of interconnected dead-end pores. Despite the ability of BET to reveal the changes in porosities, to our knowledge there are only limited examples where BET analysis has been applied to characterize changes caused by membrane fouling.

In the membrane field BET analysis has been applied mainly to characterize the porosities of virgin and developed membranes. In 1995 Chen *et al*.^11^ applied BET to study the internal area and accessibility of UF membranes to protein deposition. Only virgin membranes were measured and the acquired information on the surface area of the membrane samples was applied to estimate the degree of possible protein deposition. Calvo *et al*.^12^ studied structure and morphology of Cyclopore filters consisting of thin track-etched sheets of polycarbonate. More recently, BET has been applied to determine meso-pore distributions of molecularly imprinted polymer and reference membranes with varying cellulose acetate–sulfonated polysulfone compositions^13^; to measure average pore sizes of hollow fiber membranes^14^ and the specific surface area of pH-responsive nanofiber membrane^15^; to study the effect of silver nanoparticle grafting on porosity of sulfonated polyethersulfone membranes^16^; and to investigate the effect of the additive amount and pressurization on the surface area and pore volume of clay-hyperbranched epoxy/polyphenylsulfone nanocomposite membranes^17^.

BET has also been adapted to investigate the structure–property relationship of various kinds of polyvinylidene fluoride (PVDF) membranes. It has been used to measure the specific surface area, pore volume, and pore size distribution of PVDF membranes^18^; to measure the specific surface area of membranes with structures ranging from dense to highly asymmetric morphologies^19^; to determine pore size distribution and pore structure of polyvinylpyrrolidone grafted UF membranes^20^; and to study effects of TiO_2_ loadings on the nanostructure of the coating layer of membranes^21^.

When pore size distribution results obtained by BET have been compared to results obtained by other independent methods the results have agreed well. Pore size distribution of cellulose triacetate forward osmosis membrane was characterized with solute rejection measurements, transmission electron microscopy (TEM) and BET, and the obtained mean radius was about 0.25–0.30 nm in all of the cases^22^. Pore size of the Nadir UH004 PES membrane was measured by solute rejection, atomic force microscopy (AFM) and BET. The data obtained from different measurements suggested that the pore size was around 1 nm (solute rejection ~1.03 nm, AFM ~1.29 nm and BET ~0.86 nm)^23^.

Related to the membrane fouling investigations conducted in this study, Xiong *et al.* applied BET to characterize the structures of cake layers formed on a polymeric mesh. The cake layers were sampled at different times and cut into horizontal slices representing three layers: the top layer was in contact with the activated sludge, the bottom layer was near the mesh and the middle layer was in between. Porosities of the layers were analyzed separately and based on the results, the specific pore volume and surface area displayed an initial decline due to the disappearance of the small diameter pores (1–10 nm) with time. It was found that the large diameter pores (30–250 nm) comprised the majority of the cumulative pore volumes. The specific pore volume and specific surface area exhibited a decline from the top layer to the bottom layer due to the blockage of pores over time and depth^24^.

Lohwacharin *et al.* applied BET to determine membrane pore blocking and porosities of cake layers formed after filtering river water with and without different preadsorption treatments. The average pore diameter and the cumulative pore volume were determined for the skin layer of the virgin regenerated cellulose membrane (with MWCO of 100 kDa) and for the formed fouling cake layers together with the attached skin layers. The acquired average pore diameter of the virgin membrane (~16.3 nm) agreed well with the previously reported values. Membrane fouling was shown to decrease the average pore diameter and to increase the cumulative pore volume. Moreover, it was shown that the porosity of the cake formed dependent on the adsorbent used prior to the filtration^25^.

Among gas adsorption and desorption measurements variety of other techniques are commonly applied to estimate porosities and pore size distributions of membrane samples (see Supplementary Tab. S1 online). These techniques can be classified as equilibrium, transport or flow measurements. The main advantage of the equilibrium methods (including BET) is that they are able to measure the dead-end pores, unlike flow and transport measurements. Another strength of the gas adsorption and desorption method over the other techniques is its ability to measure the pores also in the micro-pore region, i.e. below pore size of 2 nm. Porosities can be determined also by scanning or transmission electron microscopy imaging, but due to the resolution and contrast limitations pores with size of less than few nanometers cannot be usually detected^26,27^.

In this study, BET was used in the analysis of membrane fouling caused by wood compounds of biorefinery and pulp mill streams. Fouling experiments were carried out with ultrafiltration membranes made of polysulfone and polyethersulfone, which are common membrane materials for membrane applications in pulp mills and biorefineries. Black liquor (BL), thermomechanical pulping process water (TMP) and pressurized hot-water extract (PHWE) are relevant solutions to be treated with membranes from a pulp mill/biorefinery perspective and were used as exemplary case solutions. Filtrations were conducted to a comparable volume reduction (VR) of 88% or continued until the performance of the membrane was deteriorated to a stable level (if the target VR was not reached before). The degree of irreversible fouling was evaluated by pure water permeability measurements. BET nitrogen adsorption and desorption analysis was applied to characterize the effect of fouling on the pore size distribution, pore volume and surface area. Infrared spectroscopy and contact angle analysis were used as complementary fouling characterization tools, in order to explore the fouling mechanisms and to gain further knowledge on the chemical compositions and structures of the fouling layer formed in the different processes.

## Results

### Membrane fouling characterization with BET

The commercial polymeric membranes used in this study have an asymmetric structure, consisting of a thin skin layer and a thick supportive layer. Due to this, pores from both of these layers are present in the studied membrane samples and pore volumes of skin layer and support material together form the total pore volume. A possible foulant layer might be porous as well. Selective pores of the studied ultrafiltration membranes have pore sizes ranging from 1 nm to several nm^28^. Thus the fouling induced changes both in the micro-pore (<2 nm) and meso-pore region (2–50 nm) were of interest.

**Figure 1:**
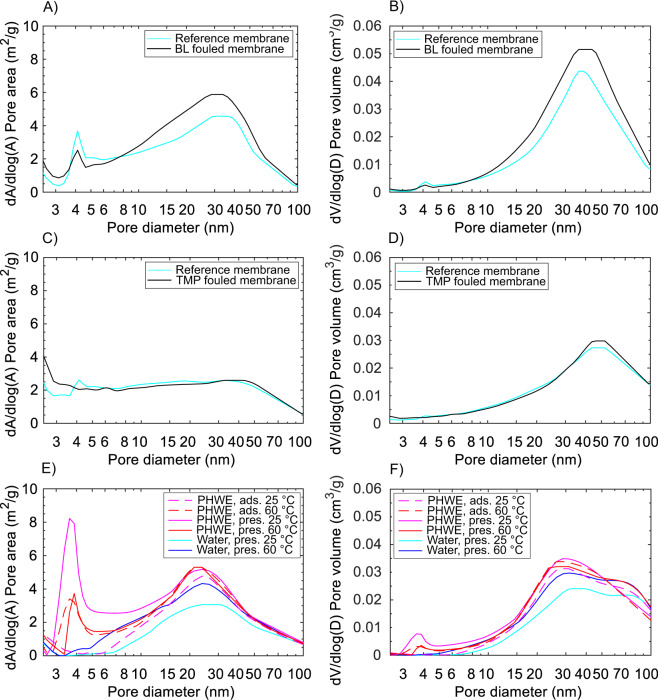
BET pore area (**A**) and pore volume (**B**) distributions of a GR95PP membrane fouled with black liquor (BL), pore area (**C**) and pore volume (**D**) distributions of a UFX5-pHt membrane fouled with thermomechanical pulping process water (TMP) and pore area (**E**) and pore volume (**F**) distributions of UP010 membrane fouled with pressurized hot-water extract (PHWE) in constant pressure and adsorptive fouling experiments at 25 and $6{0}^{\circ }$C. Desorption branch of the isotherm was used to calculate the distributions.

Adsorption isotherm reveals information about the adsorption capacity of the material. The shape of the measured nitrogen adsorption and desorption isotherms (see Supplementary Fig. S2–4) resembles a type IV isotherm with the characteristic hysteresis loop appearing in the multilayer range^29^. The shape of the hysteresis loop was most likely caused by capillary condensation taking place in the mesoporous structures. Further information about the porosity as a function of pore diameters or as a total pore volume of the whole pore size distribution was calculated from the isotherm data by the means of the BET analysis. Table 1 shows acquired cumulative BET surface area, cumulative pore volume, average desorption pore size and fraction of micro-pores. Because these cumulative or averaged values do not reveal from which kind of pores the generalized changes originate, pore volume and pore area distributions of the measured samples are also presented in Fig. 1 and differences in the porosity distributions between reference samples and fouled samples are highlighted in Supplementary Fig. S5.

**Table 1:** Surface area, cumulative pore volume and average desorption pore sizes based on nitrogen adsorption–desorption isotherms. Estimated micro-pore volume and micro-pore area are based on nitrogen adsorption–desorption isotherms and t-plot model. *Fractions of the micro-pores were too low to be determined.

Membrane	Experiment type	Sample name	Surface area (m^2^/g)	Pore volume (cm^3^/g)	Desorption pore size (nm)	Micro-pore volume (cm^3^/g)	Micro-pore area (m^2^/g)	Flux decline (%)
GR95PP (2 kDa PES)	Constant pressure	Pure water	5.5	0.029	27.7	0.00016	0.58	
		Black liquor	6.5	0.037	27.3	*	*	$-$28
UFX5pHt (5 kDa PSU)	Constant pressure	Pure water	6.1	0.025	23.2	*	*	
		TMP process water	6.2	0.027	21.7	*	*	80
UP010 (10 kDa PES)	Constant pressure	Water at pH 3.5 ($2{5}^{\circ }$C)	4.7	0.021	17.9	0.00018	0.53	
		Water at pH 3.5 ($6{0}^{\circ }$C)	5.3	0.026	19.8	0.00012	0.44	
		PHWE ($2{5}^{\circ }$C)	6.0	0.031	20.7	*	*	34–60
		PHWE ($6{0}^{\circ }$C)	5.4	0.029	21.4	*	*	100
	Adsorption	PHWE ($2{5}^{\circ }$C)	5.2	0.026	20.3	0.000051	0.29	22–27
		PHWE ($6{0}^{\circ }$C)	5.1	0.029	22.7	*	*	100

#### Porosity changes after fouling with BL

An increase of 18% in BET surface area and an increase of 28% in cumulative pore volume were found for the GR95PP membrane after fouling with BL (Table 1). However, the average pore size experienced a small decrease of less than 2%, that probably stems from the blockage of the widest pores of the membrane. In addition, the total micro-pore volume was reduced significantly. Figure 1 shows the pore area (A) and pore volume (B) distributions of the reference membrane and fouled membrane. Because the number of pores present at the certain pore diameter determines the surface area at that particular pore diameter, a decrease in pore volume at the certain pore width comes along with a decrease of the pore area at the same pore width (see Supplementary Fig. S5). Correspondingly, an increase in pore volume at the certain pore width comes along with an increase in the pore area at the chosen pore width. This can be seen when comparing the pore volume and pore area distributions. When comparing the distributions of the conditioned membrane with the fouled one it can be seen that the pores with diameters of 4.5–8 reduced their pore volume and area as the result of the fouling. It is possible that the wider pores are getting filled with larger molecules resulting in a decrease of available pore volume and decrease of average pore size. In contrast to that, smaller pores (< 3.8 nm) experienced a small increase in pore volume but a relatively great increase in pore area. This could originate from cake layer build up. A sharp decline in pore area at an almost constant pore volume can be seen for pores with a diameter of around 5 nm.

#### Porosity changes after fouling with TMP

An increase of less than 2% in BET surface area was found for the UFX5-pHt membrane from a conditioned to a fouled membrane’s state (Table 1). The cumulative pore volume underwent a slight increase of 7%. The average pore size decreased about 7% after fouling with TMP process water. Based on the pore volume and pore area distributions of the conditioned and the fouled UFX5-pHt membrane, the decrease in average pore size originates from the reduction of pores with diameters between 5–30 nm (Fig. 1). However, wider pores experienced an increase in pore volume and a slight increase in pore area which could presumably origin from cake layer formation by bigger molecules. Moreover, it was found that the area but also the volume of the smaller pores with diameter below 4 nm increased as the result of the fouling (see Supplementary Fig. S5).

#### Porosity changes after fouling with PHWE

Increases of 28% and 11% in BET surface area were found for the UP010 membrane after fouling at $2{5}^{\circ }$C in constant pressure and adsorptive fouling experiments, respectively (Table 1). At $6{0}^{\circ }$C, the surface area remained almost the same after constant pressure fouling and decreased slightly after adsorptive fouling. However, the cumulative pore volume increased in all of the cases as the result of fouling: 48% and 24% in constant pressure and adsorption experiments at $2{5}^{\circ }$C, respectively, and 12% both in constant pressure and adsorption experiments at $6{0}^{\circ }$C. Smaller increases in the surface areas and pore volumes after fouling at $6{0}^{\circ }$C may origin from the formation of a less porous fouling layer than at $2{5}^{\circ }$C. The average desorption pore size increased in all of the cases: 16% and 13% after constant pressure and adsorptive fouling at $2{5}^{\circ }$C, respectively, and 8% and 15% after constant pressure and adsorptive fouling at $6{0}^{\circ }$C, respectively.

The desorption pore volume distribution (Fig. 1 and Supplemetary Fig. S5) shows, that the pore volume increased at pore sizes between 3–6 nm after cosntant pressure fouling at $2{5}^{\circ }$C and after adsorptive and constant pressure fouling at $6{0}^{\circ }$C. The pore area of these small pores increased for the same cases. Only for adsorptive fouling at 25 °C the pore volume remained almost unchanged and the area only slightly increased. The fouling increased the pore volumes and areas at 25 °C for all pores in the range of 5–70 nm. For the constant pressure fouling, the increase in pore volume was stronger than the increase in area in the bigger pores (15–70 nm) but eventually dropped. The biggest pores (>70 nm) experienced a decrease in pore volume. Fouling at 60 °C slightly decreased pore volume and are between 6.5 nm and 15 nm. However, an increase in porosity was observed and could result from porosity of the formed fouling layer or pore roughening due to adsorption of small extractives in the smaller (3–6 nm) pores. Besides fouling, also filtration at higher temperature altered the porosity of the UP010 membrane by increasing both pore volume and surface area. Volume and area of the micro-pores decreased as the result of adsorptive fouling at $2{5}^{\circ }$C. Constant pressure fouling at $2{5}^{\circ }$C and both adsorptive and constant pressure fouling at $6{0}^{\circ }$C decreased the amount of the micro-pores so much that their fraction was too small to be detected.

#### Reproducibility of the BET results

UFX5pHt and GR95PP membranes were analyzed three times in order study the reproducibility of the results. For UFX5pHt membrane, the pore size decreased slightly with each run in the BET analyzer. However, quite similar results in terms of BET surface area, pore volume and BJH pore size were found when comparing different runs of UFX5pHt membrane samples. In contrast to the results of the UFx5pHt membrane, for GR95PP membrane, the BET surface area as well as the pore size increased with each run in the BET analyzer. It has to be considered that membrane samples tend to have relatively low surface areas letting them adsorb rather low amounts of gas. The masses of the samples ($\sim 0.3$–0.7 g) and resulting surface areas ($\sim 2$–4 m${}^{2}$) were rather low which might have increased the measurement error. The sensitivity can be increased by increasing the amount of the sample or by using filler rods that decrease the free space. An alternative approach for increasing the accuracy is to change the analysis gas from nitrogen to krypton as this allows the analysis of surface areas as low as $0.5\ {{\rm{m}}}^{2}$^30^.

### Fouling induced changes in infrared spectra and contact angles

**Figure 2:**
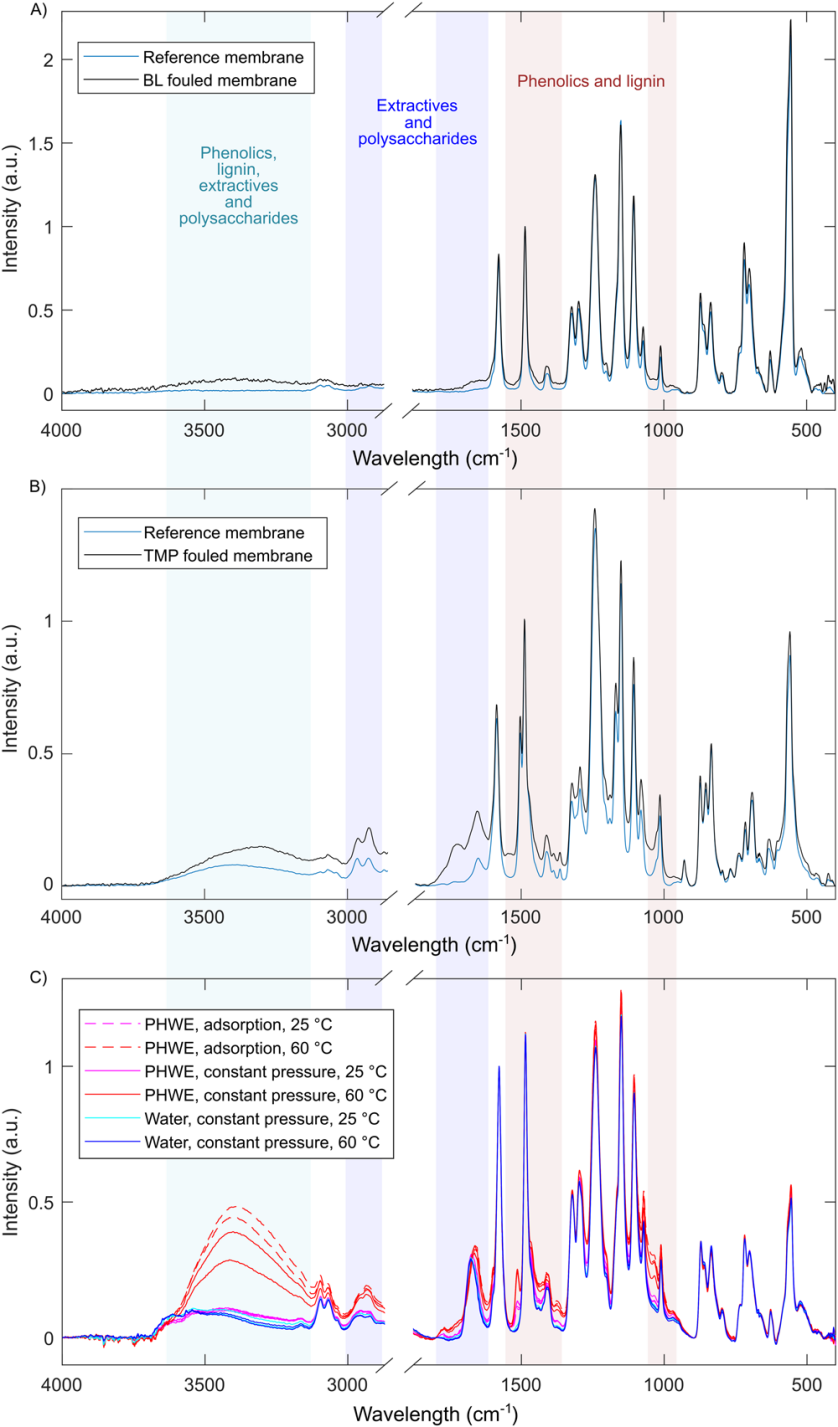
ATR-FTIR spectra of (**A**) a conditioned GR95PP membrane and a membrane fouled with black liquor (BL), (**B**) spectra of a conditioned UFX5-pHt membrane and a membrane fouled with thermomechanical pulping process water (TMP) and (**C**) spectra of UP010 membrane and a membrane fouled with pressurized hot-water extract (PHWE) in constant pressure and adsorptive fouling experiments.

Because BET does not provide any information on the chemical nature of the foulants, ATR-FTIR spectroscopy and contact angle analysis were applied as complementary fouling characterization tools. Figure 2 shows the IR spectra of reference membranes and fouled membranes. The reference spectra of the GR95PP and UP010 membranes can be assigned to characteristic PES membranes. The reference spectrum of the UFX5-pHt contains peaks characteristic for PSU membranes and are in accordance to Thuvander *et al*.^31^ who investigated the same membrane.

#### Chemical characteristics of BL fouling layer

A broad O–H stretching peak can be seen in spectrum of the GR95PP membrane fouled with BL at the area between 3100 and 3600 ${{\rm{cm}}}^{-1}$ and could be caused by water, hydroxyl groups of polysaccharides or by phenolic groups of ligneous compounds and extractives^32–34^. The impact of water to the O–H stretching can be excluded as all of the samples were stored in a desiccator for drying. The areas between the peaks 1320 ${{\rm{cm}}}^{-1}$ and 1410 ${{\rm{cm}}}^{-1}$; 1410 ${{\rm{cm}}}^{-1}$ and 1485 ${{\rm{cm}}}^{-1}$ as well as 1485 ${{\rm{cm}}}^{-1}$ and 1576 ${{\rm{cm}}}^{-1}$ are intensified as well and can be attributed to skeletal vibration of phenolic compounds such as lignin^35^. Moreover, a new area around 1650 ${{\rm{cm}}}^{-1}$ appeared which can be assigned to carbonyl containing components such as natural lignin or terpenoid extractives.

The contact angle changed from 54${}^{\circ }$ after conditioning to 46${}^{\circ }$ after fouling with BL indicating a more hydrophilic membrane surface supporting the fouling caused by ligneous compounds.

#### Chemical characteristics of TMP fouling layer

The O–H stretching area was slightly intensified for the UFX5-pHt membrane fouled with TMP process water. This intensification likely origins from fouling of polysaccharides such as hemicelluloses^31,36^. The peaks at 1014 and 1080 ${{\rm{cm}}}^{-1}$ are attributed to the aliphatic C–C and aromatic –CH rocking which is assigned to polysaccharides. The peak at 1736 ${{\rm{cm}}}^{-1}$ can be assigned to the acetyl groups of polysaccharides and to extractives. The peak at 2964 ${{\rm{cm}}}^{-1}$ is attributed to the aromatic C–H stretching of polysulfone as well as polysaccharides. Both are intensified for the fouled membrane.

The contact angle changed from 71.5${}^{\circ }$ after conditioning to 39.5${}^{\circ }$ after fouling with TMP process water indicating that the surface fouling was caused by hydrophilic compounds.

#### Chemical characteristics of PHWE fouling layer

Neither adsorptive nor constant pressure fouling of UP010 membrane at $2{5}^{\circ }$C lead to significant increases in intensities in the O–H region. However, both adsorptive and constant pressure fouling at $6{0}^{\circ }$C caused remarkable intensity increases in O–H and C–H peaks, and interestingly, the increase in intensity was higher after adsorptive fouling. Peaks at 2850–3000 ${{\rm{cm}}}^{-1}$ (alkyl C–H stretchings and O–H stretchings of carboxylic acids) may originate from fatty acids, resin acids, galacturonic acid and xylan. The spectra show fouling originated peaks which can be assigned to phenolics and lignin at 1035 ${{\rm{cm}}}^{-1}$, 1375 ${{\rm{cm}}}^{-1}$, 1465 ${{\rm{cm}}}^{-1}$, 1425 ${{\rm{cm}}}^{-1}$ and 1514 ${{\rm{cm}}}^{-1}$. Based on the intensities, fouling caused by phenolics and lignin was about twice more severe at $6{0}^{\circ }$C than at $2{5}^{\circ }$C. Based on the IR data, adsorptive fouling caused by phenolics and lignin was slightly less intense than constant pressure fouling at $2{5}^{\circ }$C, but as heavy as constant pressure fouling at $6{0}^{\circ }$C. Peaks at 1640–1680 ${{\rm{cm}}}^{-1}$ originate from conjugated C=O stretching and peaks around 1710–1780 ${{\rm{cm}}}^{-1}$ can be assigned to C=O stretchings in unconjugated carbonyl groups of extractives and xylanes. It is notable, that a carbonyl peak of the virgin membrane at 1676 ${{\rm{cm}}}^{-1}$ shifted to 1671 ${{\rm{cm}}}^{-1}$ after fouling at $2{5}^{\circ }$C and further to 1663 ${{\rm{cm}}}^{-1}$ after fouling at $6{0}^{\circ }$C indicating interactions between membrane polymers and foulants.

Both adsorptive and constant pressure fouling of UP010 membrane at $2{5}^{\circ }$C decreased the contact angle from 46${}^{\circ }$ to 41${}^{\circ }$ while at $6{0}^{\circ }$C the contact angles increased to 49${}^{\circ }$. The hydrophilization of the membrane at $2{5}^{\circ }$C probably stems from the fouling caused by phenolic and ligneous compounds^37^. The hydrophobization of the membrane at $6{0}^{\circ }$C could be explained by more intensive fouling caused by the extractives which is supported by the IR spectra. Temperature and pH have a significant effect on deposition tendency of wood extractives and on interactions between the components. At the pH of the spruce extract (pH = 3.5) the resin acids and fatty acids are in undissociated form and have thus a low solubility. The increase in temperature increases the proportion of soluble undissociated resin acids which has been shown to increase deposition due to decrease in resin viscosity.^38–40^ The different kind of orientation of foulants in different conditions might also explain the observed opposite changes in contact angle values at different temperatures. Colloidal foulants have an ability to undergo molecular reorientation as the result of changes in their environment.^41,42^

## Discussion

The aim of our study was to investigate what type of information BET nitrogen adsorption and desorption technique could provide with regard to fouling of polymeric ultrafiltration membranes. The membranes were fouled with wood-originated solutions containing lignin, polysaccharides and extractives which are known as organic substances causing complex fouling processes. In general, the formation of a fouling layer on the surface of the pores and on the surface of the membrane lead to an increase in cumulative pore volumes and pore areas. Pore-blocking could be detected by estimated micro-pore results acquired by the t-plot method. In this study, a distribution of micro-pores was not measured because such measurements would have taken significantly longer time than the applied measurements in the meso-pore area. Because BET can provide only structural information on the properties of the fouling layer, pure water permeability determinations, ATR-FTIR spectroscopy and contact angle measurements were applied as complementary fouling characterization methods. The combination of BET and IR characterization enabled both density and chemical composition of the fouling layer to be estimated.

Filtration of BL lead to an increase in BET surface area and in cumulative pore volume. However, the average pore size decreased slightly. Thus, the results could be explained by adsorption of bigger molecules on pore walls of the widest pores and by formation of a porous fouling layer (see Supplementary Fig. S5). Polysaccharides such as hemicelluloses (molecular weight of 10–20 kDa) and lignin (5–10 kDa) have a size that would allow that kind of fouling. Fouling decreased pore areas and volumes also in the region of 4.5–8 nm. Here, extractives and degradation compounds of lignin and polysaccharides as smaller molecules are likely to adsorb inside these smaller pores. Wood extractives such as sitosteryl ester or glyceryl trilinoleate, representing the common wood extractive groups of steryl esters and triglycerides, with dimensions of 0.4 nm in width and 2.6 nm in length for sitosteryl ester and 1.5 nm in width and 3.2 nm in length for glyceryl trilinoleate (based on models generated at MolView v2.4). Dry Kraft lignin has a size of 1–3 nm in width and 5–9 nm in length^43^. Hence, extractives as well as lignin and its degradation compounds would be able to enter these pores and could cause pore blocking or some kind of pore smoothing which would result in a decline in pore area while the pore volume would be altered only slightly.

In pores with diameters in the range of 2.7–4.5 nm, a notable increase in the pore area is observed but the pore volume did almost not change (see Supplementary Fig. S5 online). Wood extractives such as sitosterol, oleic acid and abietic acid, representing the common wood extractive groups sterols, fatty acids and resin acids, have dimensions around 0.5 nm in width and 1.5 nm in length^2^. As an alternative to cake layer formation, it could be speculated that these extractives enter the pores causing pore roughening and hereby increase the surface area of these pores much stronger than they would increase the pore volume.

Remarkable changes in porosities together with less intense foulant peaks in IR spectra suggest that the formed fouling surface is relatively porous and the amount of foulants is lower when compared to the other cases. Based on the BET results, the micro-pore volume reduced significantly after fouling with BL even though the irreversible flux decline ratio was $-28$% (Table 1). The improvement in pure water permeability is supported by increase in hydrophilicity and could be explained by fouling caused by the phenolic compound lignin present in the BL^37,44^ or by the alkalinity of the BL solution^45^. Thus, in the case of fouling caused by BL monitoring the change in pure water permeability is not a sufficient method to reveal whether the membrane has been fouled or not.

Fouling with TMP process water increased the BET surface area and the cumulative pore volume. For pores above a diameter of 30 nm, the pore areas and volumes increased (see Supplementary Fig. S5 online). This increase could stem from cake fouling caused by hemicelluloses or extractives-polysaccharide complexes. In pore sizes between 4.5–30 nm, the pore area and the pore volume remained almost unchanged. A notable reduction in pore area and less strong reduction in pore volume can be found at 5 nm. It is possible that smaller polysaccharides or ligneous compounds from the TMP process water adsorbed into pores and by that alteredthe pore area due to smoothing but only slightly affected the pore volume. A notable increase in pore area can be found for pores in the range of 3–5 nm while the pore volume remained almost unaffected. This was also observed for the BL fouling and could thus potentially be caused by the adsorption of small extractives from the group of sterols, resin acids and fatty acids in the pores causing pore roughening.

Compared to the case of BL fouling, the intensities of the foulant peaks are higher, but the cumulative pore volume increased less. Thus, the formed fouling layer from TMP process water is probably denser than in the case of fouling caused by BL. Pore-blocking of UFX5-pHt membrane could not be detected by BET. Based on the irreversible flux decline ratio of 80% the fouling was severe. This is in accordance to previous results observed by Thuvander *et al*. They suggested that this was most likely caused by adsorptive fouling of polysaccharides^31^.

Adsorptive and constant pressure fouling caused by spruce PHWE increased the cumulative pore volume both at $2{5}^{\circ }$C and $6{0}^{\circ }$C. However, the cumulative surface area increased only in fouling experiments conducted at $2{5}^{\circ }$C. Thus, it seems that a denser fouling layer is formed at $6{0}^{\circ }$C than at $2{5}^{\circ }$C. It is likely that this layer is composed of a mixture of polysaccharides, extractives and ligneous compounds. It should be noted that the fouling experiment with TMP process water was similarly conducted at high temperature ($7{0}^{\circ }$C) and the resulting fouling layer was presumably relatively dense as well. The differences in the formation of the fouling layer at different temperatures probably originate from the changes in the solubilities of the foulants, particularly from the changes in the behavior of colloidal extractives. The irreversible flux decline ratios for the membranes fouled with PHWE at $2{5}^{\circ }$C were 22–27% and 34–60% after adsorptive and constant pressure fouling, respectively (see Supplementary Fig. S1). At $6{0}^{\circ }$C, the membrane got completely plugged both in adsorptive and constant pressure experiments and the irreversible flux decline ratio was 100%. Reductions in the amount of micro-pores were in accordance with the irreversible flux decline ratios. Koivula *et al*. reported similarly, that filtration of wood extract with commercial PES and PSU membranes was almost impossible due to severe and rapid fouling caused by ligneous compounds^1,2^.

Overall, based on the increases in porosities in the meso- and/or macro-pore regions the development of the fouling layer reached the stage of gel or cake layer built-up in all of the studied cases. The fouling layer structures caused by BL and PHWE at $2{5}^{\circ }$C were more porous while the fouling layers caused by TMP at $7{0}^{\circ }$C and PHWE at $6{0}^{\circ }$C were considered denser. Adsorption and deposition of lignin, polysaccharides and extractives on the pore walls and on the surface of the membranes can be regarded as the initial fouling mechanisms in all of the cases but the domination of the foulant species varied.

## Materials and Methods

### Membrane and fouling layer characterization methods

#### Brunauer-Emmett-Teller (BET) analysis

Gas adsorption and desorption analyses were done with Micromeritics 3Flex surface characterization analyzers and the Smart VacPrep 067 degassing units. The membrane samples were cut into small pieces and degassed at $5{0}^{\circ }$C for 4–12 hours prior to the analysis. Adsorption and desorption isotherms were measured with nitrogen gas. Ultrafiltration membranes have pore sizes in the range of 1–10 nm^46^. Hence, in this study meso-pore analysis was performed. MicroActive software was applied to calculate the results from the measured isotherms. Pore volume and pore area distributions were attained using the Barrett, Joyner, and Halenda (BJH) method^47^ and the reference curve of Harkins-Jura^48^. The desorption branch was used for the calculations. The total micro-pore volume was obtained by the t-plot method. A more detailed summary on BET theory for characterization of membrane samples can be found in the Supplementary Information.

#### Infrared spectroscopy

Attenuated total reflectance Fourier transform infrared spectroscopy (ATR-FTIR) was applied to measure the amount of lignin originated fouling. GR95PP and UFX5-pHt membranes fouled with BL and TMP process water, respectively, were analyzed using Bruker ALPHA-p FTIR spectrometer in ATR mode. The spectral range was 500–4000 ${{\rm{cm}}}^{-1}$ resolution 2 ${{\rm{cm}}}^{-1}$ and data interval 1 ${{\rm{cm}}}^{-1}$. UP010 membranes fouled with PHWE were analyzed using Perkin-Elmer Frontier spectrometer equipped with a diamond crystal. The spectral range was 400–4000 ${{\rm{cm}}}^{-1}$, resolution 4 ${{\rm{cm}}}^{-1}$ and data interval 1 ${{\rm{cm}}}^{-1}$. 10 points were measured from each membrane. Spectra were processed with ATR correction, baseline correction and normalization.

#### Contact angle measurements

Contact angles were measured by sessile drop method. Samples were dried in a desiccator before measurements. Contact angles of the GR95PP and the UFX5-pHt membranes fouled with BL and TMP process water, respectively, were measured by drop and bubble shape tensiometer PAT-1 (SINTERFACE Technologies). Contact angle was read out with the software PAT-1 (version 8.01.23). Contact angles of the UP010 membranes fouled with PHWE were measured by KSV CAM 101 instrument connected to a CCD camera. Captured images were treated with curve fitting tool in CAM 2008 software to determine the contact angles.

### Membranes

UFX5-pHt polysulfone membrane with molecular weight cut-off (MWCO) of 5 kDa (Alfa Laval) and GR95PP (Alfa Laval) and UP010 (Microdyn-Nadir) polyethersulfone membranes with MWCOs of 2 and 10 kDa, respectively, were used.

### Fouling solutions

#### Black liquor

Black liquor (BL) provided by the Smurfit Kappa Piteå mill, Smurfit Kappa, Sweden was used. The BL originates from a Kraft process where a mixture of hardwood and softwood is used. The solution contained a high concentration of lignin, 232.6 mg/g and some polysaccharides, total amount 16.7 mg/g. The pH of the solution was 13.4.

#### Thermomechanical pulping process water

Process water from thermomechanical pulping (TMP) of soft wood, mainly spruce, provided by Kvarnsveden Mill, Stora Enso, Sweden was used. The process water was relatively dilute, with a concentration of less than 1% (w/w) total solids and contained apart from hemicelluloses, residues of lignin, salts, and extractives^36^. The pH of the process water was 4.5.

#### Pressurized hot-water extract

Pressurized hot-water extract (PHWE) was prepared from spruce sawdust (provided by Mustola Timber, Finland) that had been filtered through 5.6 mm sieve. Cooking was done in a batch reactor setup at $16{0}^{\circ }$C for 2 hours at pressure of 6.5 bar. The weight ratio of water to wood was 5:1. The total carbon content of the extract was 6.9 g/L and the pH was 3.5.

### Fouling experiments

#### Fouling experiments with BL

Constant pressure fouling experiments were done using a 400 mL dead-end cell and a polyethersulphone GR95PP membrane with an active area of 32 ${{\rm{cm}}}^{2}$. Membranes were conditioned prior to the experiments with 200 mL of 0.4% (w/v) NaOH at 5.5 bar, $2{5}^{\circ }$C for 0.5 hour. The conditioned membrane was rinsed with a total of 1200 mL deionized water. Thereafter, the pure water permeability was determined at 5.5 bar and $2{5}^{\circ }$C. Constant pressure fouling experiments were performed at 5.5 bar and $2{5}^{\circ }$C. The filtration was continued until a VR of 88% was reached. Pure water permeability after fouling was determined at 5.5 bar and $2{5}^{\circ }$C.

#### Fouling experiments with TMP process water

Constant pressure fouling experiments were carried out using a 400 mL dead-end cell and the polysulphone UFX5-pHt membrane with an active area of 32 ${{\rm{cm}}}^{2}$. This type of membrane is permanently hydrophilic. The membranes were conditioned with 400 mL of 1% (w/v) Ultrasil 10 (Ecolab Deutschland GmbH) at 50 ${}^{\circ }$C for 1 hour. The conditioned membrane was thereafter rinsed with a total of 1200 mL deionized water and the pure water permeability was determined from several measurements at 0.5, 1.0, 1.5 and 2.0 bar and $2{5}^{\circ }$C. Constant pressure fouling experiments were carried out at 2 bar and $7{0}^{\circ }$C. The filtration was continued until a VR of 88% was reached. Pure water permeability after fouling was determined at 0.5, 1.0, 1.5 and 2.0 bar and $2{5}^{\circ }$C.

#### Fouling experiments with PHWE

Adsorptive and constant pressure fouling experiments were done using a 300 mL dead-end Amicon cell (Millipore, Merck KGaA) and the UP010 membrane with an active area of 38 ${{\rm{cm}}}^{2}$. Membranes were pre-wetted prior to experiments and pressurized at 3 bar for 1 hour to stabilize them. The pure water permeability was measured at 1 bar and $2{5}^{\circ }$C. The fouling experiments were conducted at 25 and $6{0}^{\circ }$C. Adsorptive fouling experiments were done without applied pressure by stirring the pressurized hot-water extract over the membrane for 2 hours. Constant pressure fouling experiments were conducted at 3 bar and the performance of the membrane declined fast at both 25 and $6{0}^{\circ }$C. At $2{5}^{\circ }$C filtration was halted at a VR of 6% (when deteriorated permeability had been stabilized for about 2 hours). At $6{0}^{\circ }$C the membrane got plugged immediately in the beginning of the filtration and no permeate could be collected, but the fouling step was continued for 2 hours. Reference experiments were done by using pure water instead of the extract in the fouling step. pH of the pure water was adjusted to 3.5 with hydrochloric acid. Pure water permeability after fouling was determined at 1 bar and $2{5}^{\circ }$C. Stirring rate was kept at 350 rpm throughout the experiments.

## Supplementary information

Supplementary Information.

## Data Availability

The data sets analyzed during the current study are available from the corresponding author on reasonable request.
