# Supplementary material for: Analysis of membrane fouling by Brunauer-Emmett-Teller nitrogen adsorption/desorption technique
Source: Sci Rep. 2020 Feb 25;10:3427. doi: 10.1038/s41598-020-59994-1 (PMC7042297; doi:10.1038/s41598-020-59994-1)
Supplement: Supplementary file 1 — Supplementary Information. [file 41598_2020_59994_MOESM1_ESM.pdf]

Supplementary Information

Analysis of membrane fouling by Brunauer-Emmet-Teller nitrogen adsorption/desorption technique

Tiina Virtanen,[*,†] Gregor Rudolph,[‡] Anastasiia Lopatina,[†] Basel Al-Rudainy,[‡] Herje Schagerlöf,[‡] Liisa Puro,[†] Mari Kallioinen,[†] and Frank Lipnizki[‡]

[†] *LUT University, Department of Separation Science, P.O. Box 20, FI-53851 Lappeenranta, Finland*

[‡] *Lund University, Department of Chemical Engineering, P.O.Box 124, SE-221 00 Lund, Sweden*

***Corresponding author's e-mail address: tiina.virtanen@lut.fi**

Table S1: Main techniques used for characterization of membrane porosity, pore size and pore size distribution. *d* = diameter of the measurement gas or solute, SEM = scanning electron microscopy, TEM = transmission electron microscopy. Adapted from [1-4]

**Type**	**Technique**	**Information**	**Scale**	**Considerations**
Equilibrium	Gas adsorption /desorption	Pore size distribution, surface area	>*d*	Samples must be dried
	Thermoporometry	Pore size distribution, pore shape	>2 nm	Samples must be wetted
	Permporometry	Pore size distribution	>2 nm	Detects only active pores
	Bubble point	Maximum pore size	>13 nm	Not suitable for tight membranes
Transport /flow	Solute permeation	Cut-off value	>*d*	Results may be dependent on the properties of the solute
	Liquid-liquid displacement	Pore size distribution	>*d*	Detects only active pores, swelling of polymeric membranes skews results
	Mercury Porosimetry	Pore size distribution	>4 nm	Not suitable for tight membranes
Imaging	Electron microscopy	Pore size distribution, pore shape, surface porosity	>20 nm (SEM)<br>>0.2 nm (TEM)	Samples must be dried, resolution and contrast limit detection of micro-pores

[1] Bernstein, R., Kaufman, Y. & Freger, V. Membrane Characterization, 1–41 (John Wiley & Sons, Inc., 2013).

[2] Cuperus, F. & Smolders, C. Characterization of UF membranes: Membrane characteristics and characterization techniques. Adv. Colloid Interface Sci. 34, 135–173, DOI: 10.1016/0001-8686(91)80049-P (1991).

[3] Tylkowski, B. & Tsibranska, I. Overview of main techniques used for membrane characterization. J. Chem. Technol. Metall. 50, 3–12 (2015).

[4] Klobes, P., Meyer, K. & Munro, R. G. Porosity and specific surface area measurements for solid materials. Tech. Rep., NIST Recommended Practice Guide (2006).

Summary of BET theory for characterization of membrane samples

Samples need to be pretreated prior to analysis by degassing in order to remove moisture and atmospheric vapors and gases. The sample pretreatment is done by applying high vacuum and heat, because the physisorption of gases decreases with decreasing pressure and increasing temperature. Optionally, heat and flowing gas can be applied. Pretreatment must be done with care because the attempt to clean the membrane samples in too vigorous conditions might cause serious damage to membrane structure. Deformations caused by too high temperatures can be avoided by keeping the temperature below a critical value during the degassing. The critical temperature depends on the polymeric material of the membrane. If the pretreatment temperature is higher than 100 °C possible steaming, i.e. hydrothermal alteration caused by internal vapor pressure of water should be taken into account [1].

The gas adsorption analysis is done by reducing the sample temperature to the boiling point of liquid nitrogen at normal pressure (-195.8 °C). The sample tube is first evacuated and then adsorbing gas is dosed into the sample tube in controlled increments, the pressure is allowed to equilibrate and the amount of adsorbed gas is calculated. Classical gas adsorption theory assumes that gas molecules form a monolayer on the cold surface and the saturation vapor pressure is reached before a second layer starts to form. Consequently, as more gas is dosed and pressure is increased, multilayer coverage will begin with the smallest pores being filled first. Pores with a diameter less than 2 nm are called micropores and pores with diameter between 2–50 nm are referred to as mesopores. In adsorption/desorption experiments, pressure is usually expressed as the relative pressure, P/P_0, where P is the actual gas pressure and P_0 is the saturation vapor pressure of the adsorbing gas. [1]

The amount of gas adsorbed the measured pressure data are used to generate an adsorption isotherm. The desorption isotherm is acquired in a contrary way. Hysteresis between adsorption and desorption isotherms is typical for mesoporous samples which have a wide range of pore sizes. The hysteresis stems from the differences between gas condensation within the pores and gas evaporation from the pores. Density functional theory simulations have suggested that the adsorption branch comprises of metastable states, while the desorption branch represents the thermodynamic equilibrium. It is assumed that pores of the polymeric membranes in the nanometer scale consist of capillary tubes and can be modeled as cylindrical pores. [2][3] It is not advisable to use adsorption branch for cylindrical pores as the cylindrical meniscus is not stable. [1] As a result, the desorption branch is generally used for the determination of the pore size distribution and was consequently also applied in this study.

The isotherm data is typically treated with gas adsorption theories to gain the spesific surface area of a sample. The thickness of the adsorbed layer can be determined by the reference curve of Halsey, Harkins-Jura or Broekhoff-DeBoer who have derived the equations from the adsorption equation of Brunauer-Emmett-Teller. During the measurements of mesoporous samples, pores fill at pressures that are below the prevailing saturated vapor pressure of the gas due capillary condensation. This pore filling happens as the result of the attractive forces created by two opposing pore walls. Thus correlating functions need to be used to relate the pore size with a critical condensation pressure [1]. The so called BJH method [4] was developed to deal with coarsely porous adsorbents with a wide range of pore sizes and bases on the Kelvin model of pore filling. It can be applied for determining the pore size and pore volume distribution of a mesoporous samples of any nature. The BJH method is valid only in the mesopore and beginning of macropore range.

In this study, pore volume and area distributions were attained using the BJH method and the reference curve of Harkins-Jura. Because the BJH method is not valid in the micropore range, the total micropore volume was obtained by the t-plot method in combination with low pressure adsorption data below a relative pressure 0.01. The

linear region of the V_a vs. t plot is extrapolated to the y-axis of the plot. A positive intercept is equivalent to the micropore volume. When there is no clear linear region in the t-plot, the acquired micropore volume depends on the interval chosen for extrapolation. The correlation coefficient should be 0.99 or better.

[1] P.A. Webb, C. Orr, Analytical methods in fine particle technology, Micromeritics Instrument Corporation, Norcross, Georgia, USA, 1997.

[2] E. Drioli, L. Giorno, Comprehensive Membrane Science and Engineering, Elsevier, Kidlington, Oxford, UK, 2010.

[3] N.N. Li, A.G Fane, W.S.W Ho, T. Matsuura, Advanced Membrane Technology and Applications, John Wiley \& Sons, Inc., Hoboken, New Jersey, USA, 2008.

[4] E.P. Barrett, L.G. Joyner, P.P. Halenda, The determination of pore volume and area distributions in porous substances. i. Computations from nitrogen isotherms, J. Am. Chem. Soc. 73 (1) (1951) 373--380. doi: 10.1021/ja01145a126

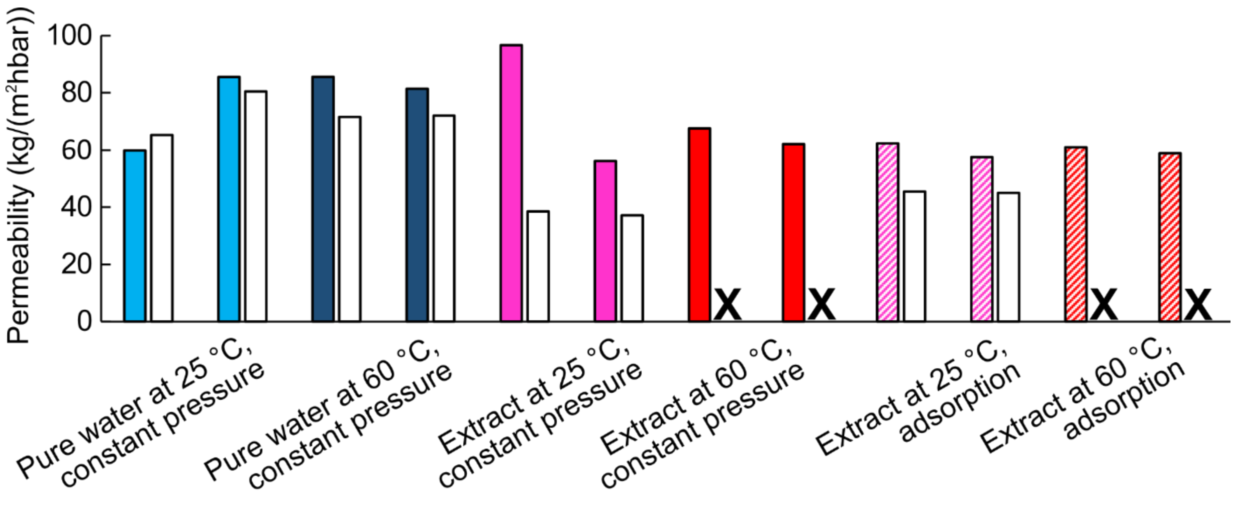

Figure S1: Pure water permeabilities measured at 25 °C before (coloured) and after (white) fouling in constant pressure and adsorption experiments of spruce hot-water extract. **X** marks are shown when pure water permeabilities could not be measured after fouling.

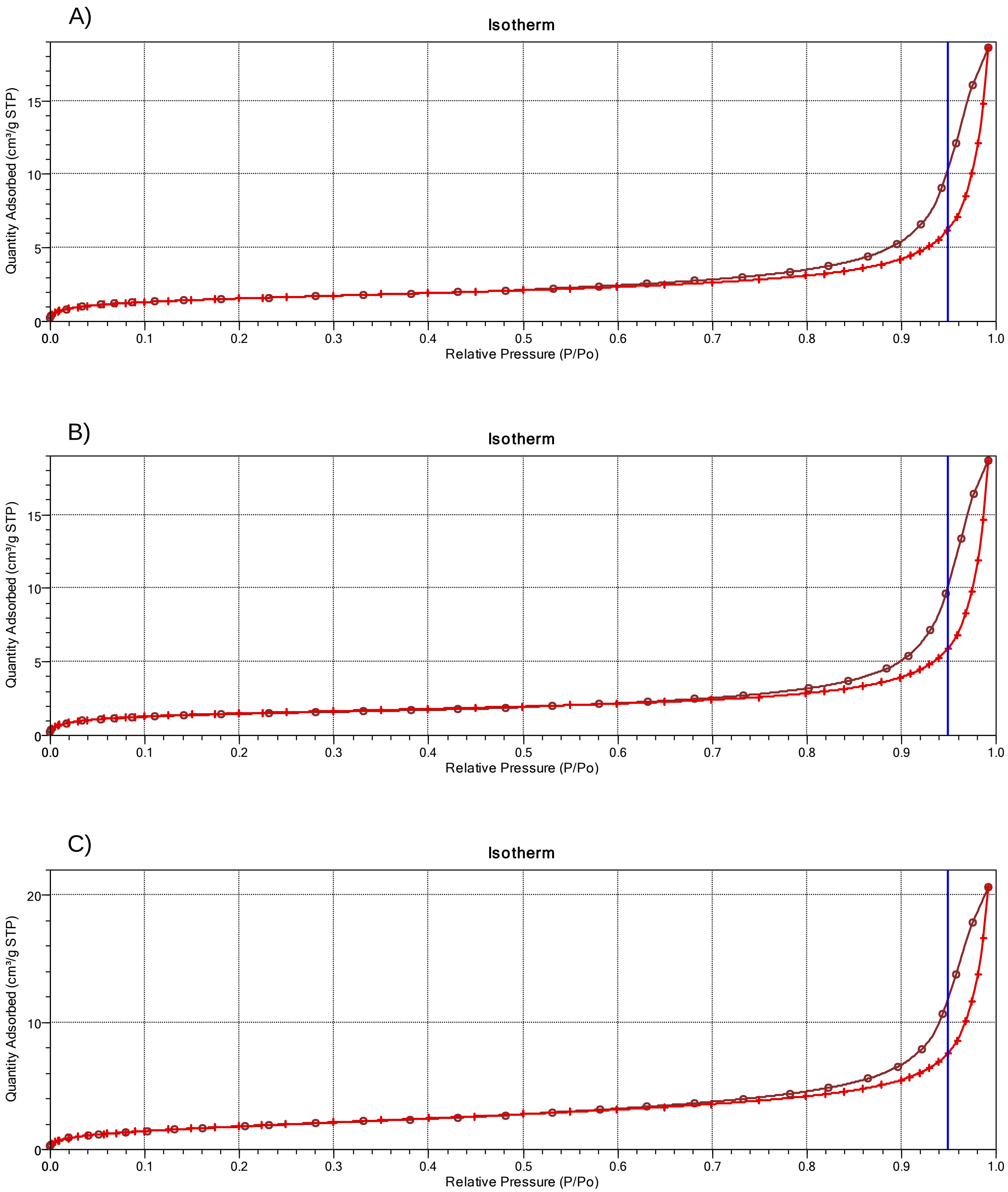
A)
Isotherm
Quantity Adsorbed (cm³/g STP)
Relative Pressure (P/Po)
B)
Isotherm
Quantity Adsorbed (cm³/g STP)
Relative Pressure (P/Po)
C)
Isotherm
Quantity Adsorbed (cm³/g STP)
Relative Pressure (P/Po)

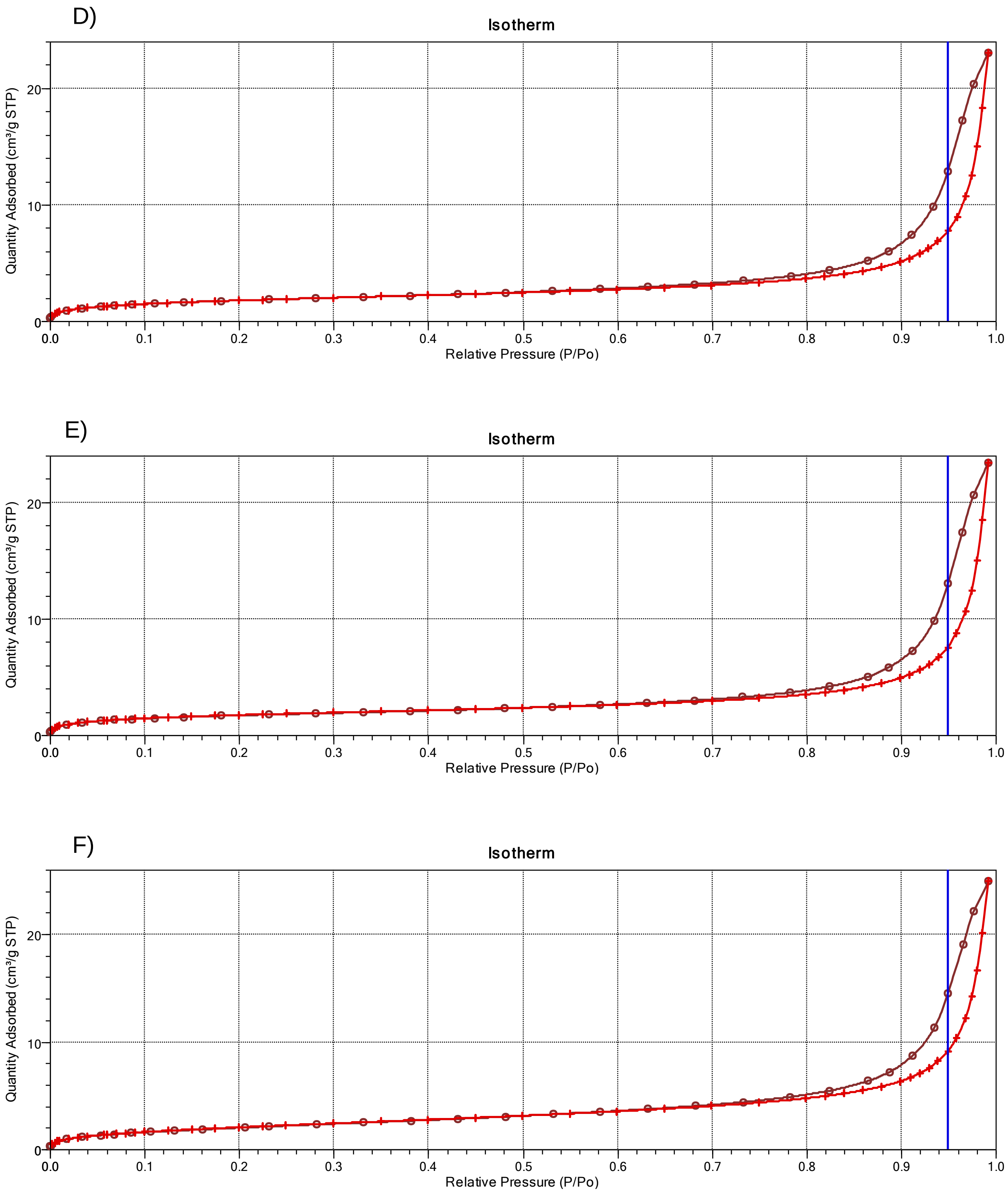

Figure S2: Isotherms of the GR95PP membranes samples fouled with black liquor. A) reference with water, B) first rerun of reference with water, C) second rerun of reference with water, D) constant pressure fouling with black liquor, E) first rerun of constant pressure fouling with black liquor, F) second rerun of constant pressure fouling with black liquor.

A)

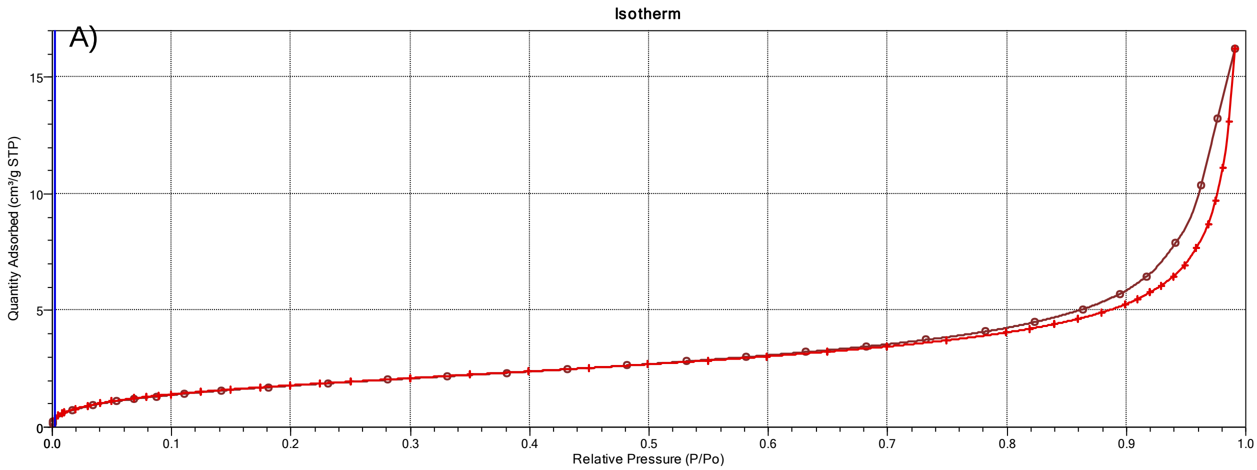

Isotherm
Quantity Adsorbed (cm³/g STP)
Relative Pressure (P/Po)
0
5
10
15
0.0
0.1
0.2
0.3
0.4
0.5
0.6
0.7
0.8
0.9
1.0

B)

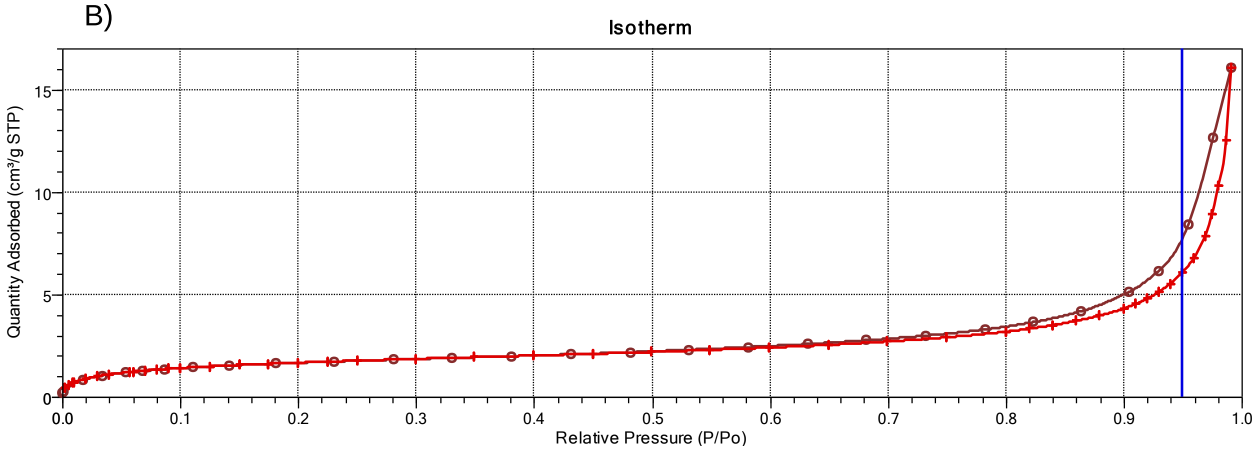

Isotherm
Quantity Adsorbed (cm³/g STP)
Relative Pressure (P/Po)
0
5
10
15
0.0
0.1
0.2
0.3
0.4
0.5
0.6
0.7
0.8
0.9
1.0

C)

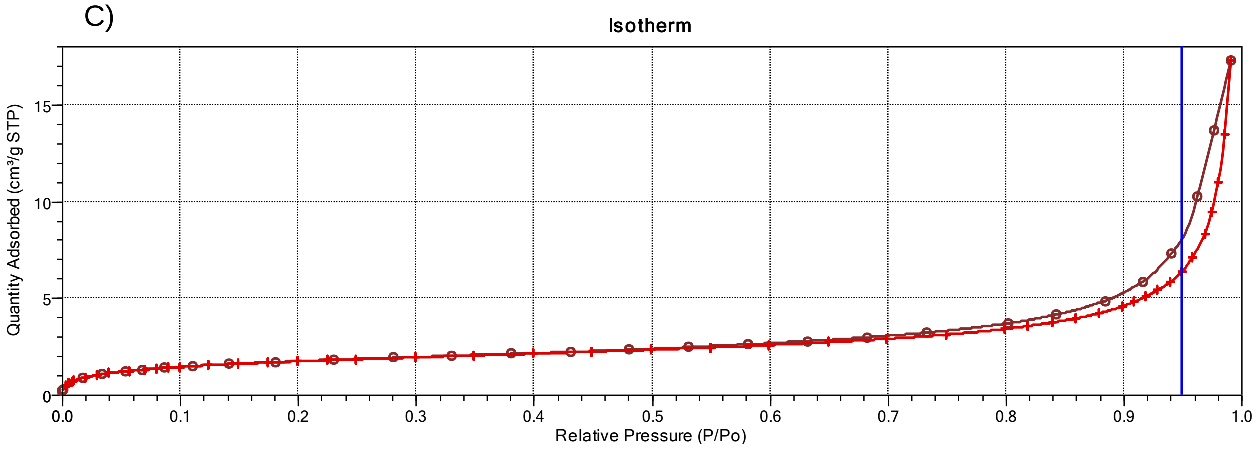

Isotherm
Quantity Adsorbed (cm³/g STP)
Relative Pressure (P/Po)
0
5
10
15
0.0
0.1
0.2
0.3
0.4
0.5
0.6
0.7
0.8
0.9
1.0

D)

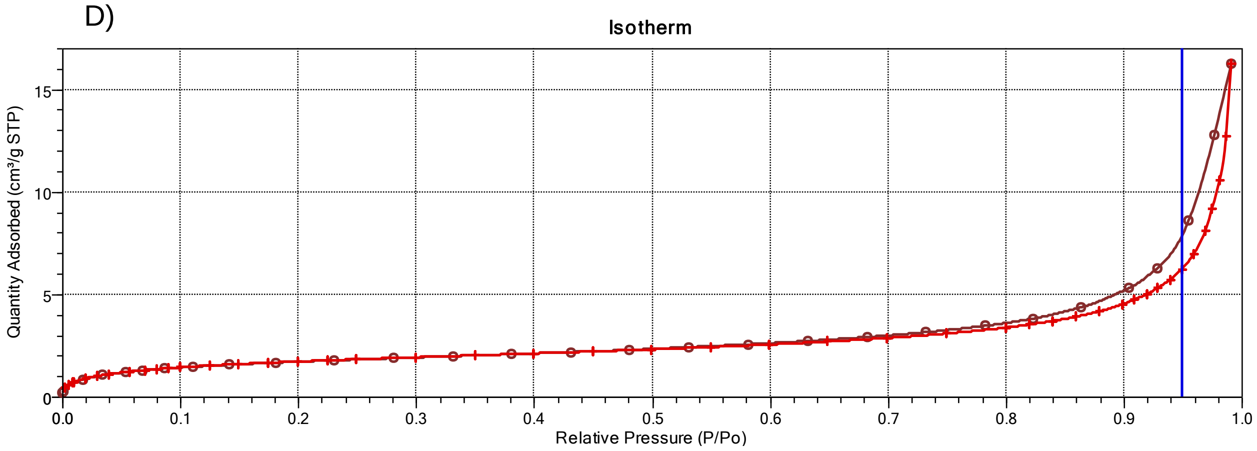

Isotherm
Quantity Adsorbed (cm³/g STP)
Relative Pressure (P/Po)

E)

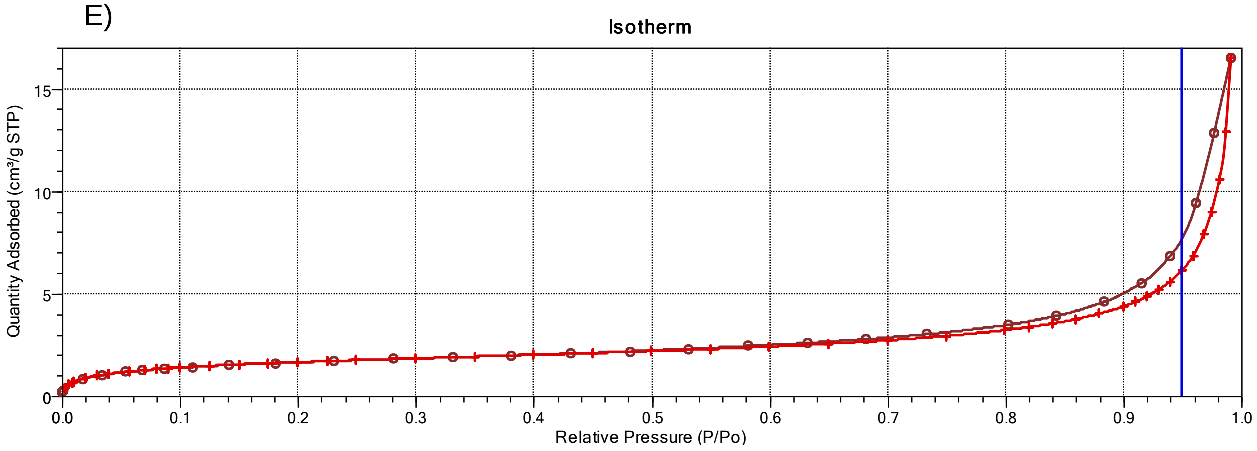

Isotherm
Quantity Adsorbed (cm³/g STP)
Relative Pressure (P/Po)

F)

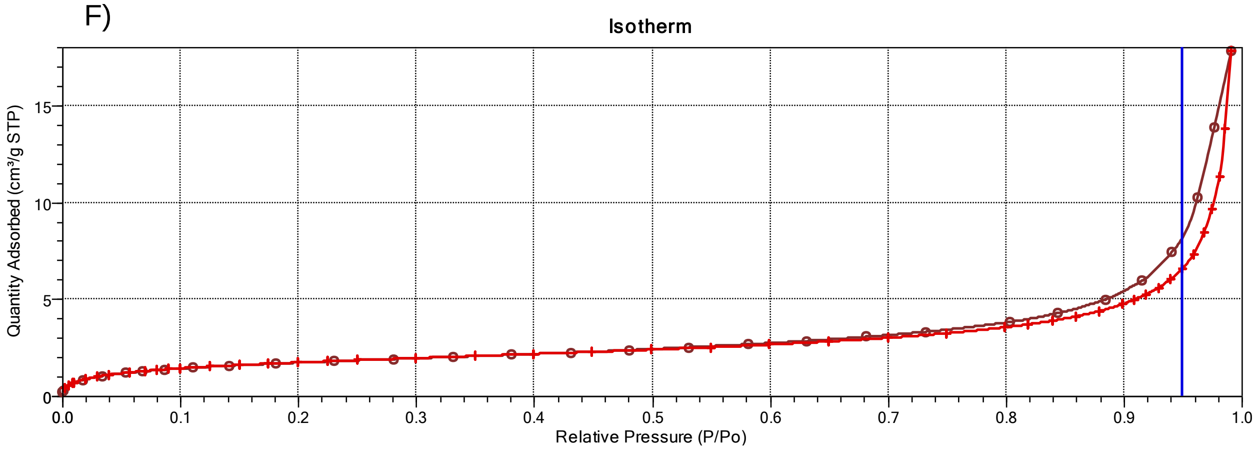

Isotherm
Quantity Adsorbed (cm³/g STP)
Relative Pressure (P/Po)

G)

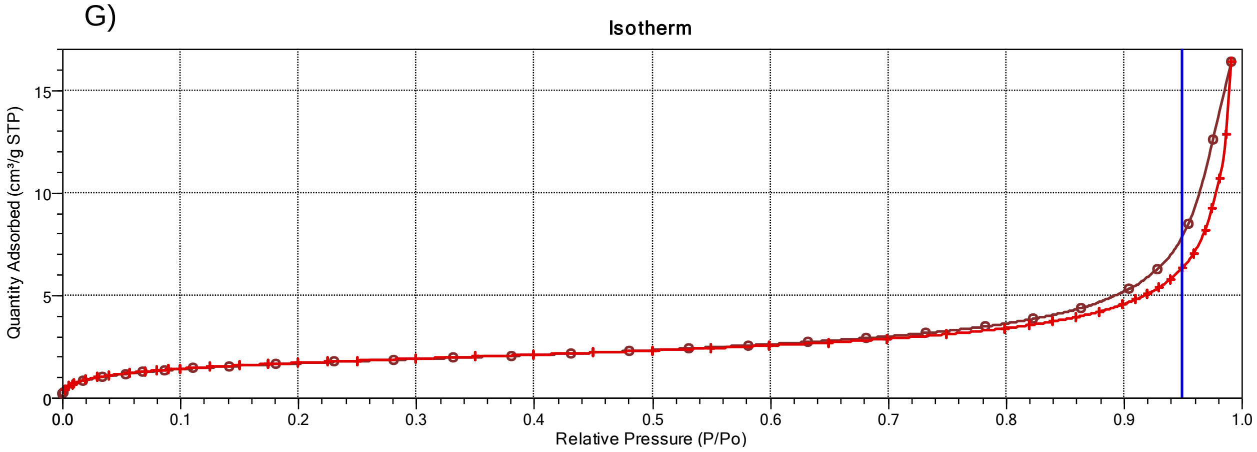

Isotherm
Quantity Adsorbed (cm³/g STP)
Relative Pressure (P/Po)
0
5
10
15
0.0
0.1
0.2
0.3
0.4
0.5
0.6
0.7
0.8
0.9
1.0

H)

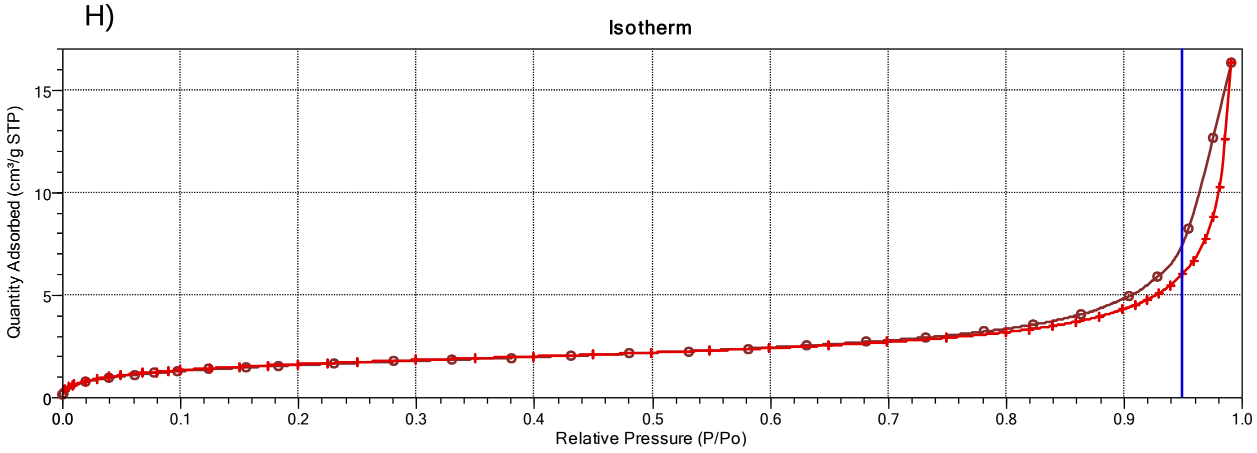

Isotherm
Quantity Adsorbed (cm³/g STP)
Relative Pressure (P/Po)
0
5
10
15
0.0
0.1
0.2
0.3
0.4
0.5
0.6
0.7
0.8
0.9
1.0

I)

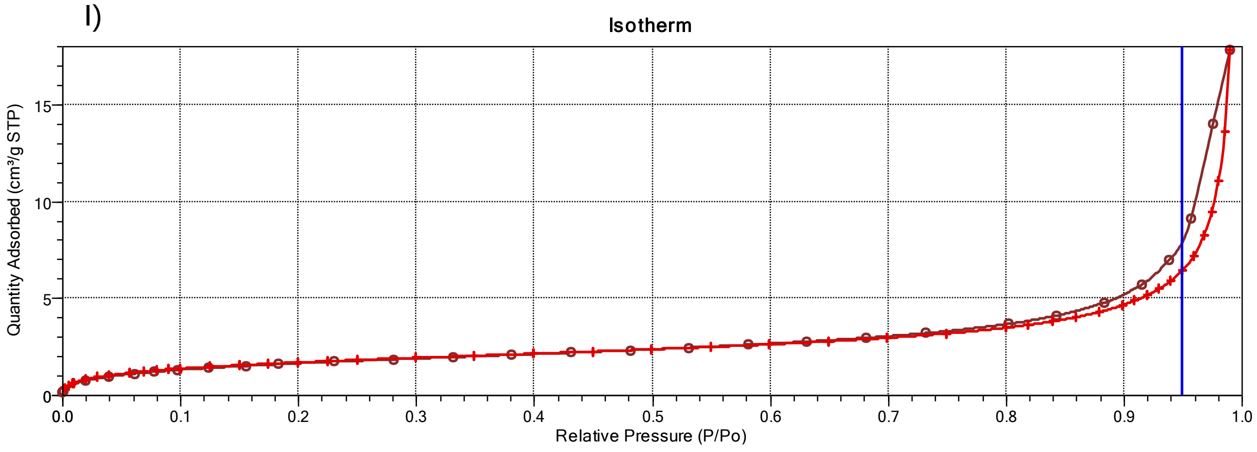

Isotherm
Quantity Adsorbed (cm³/g STP)
Relative Pressure (P/Po)
0
5
10
15
0.0
0.1
0.2
0.3
0.4
0.5
0.6
0.7
0.8
0.9
1.0

J)

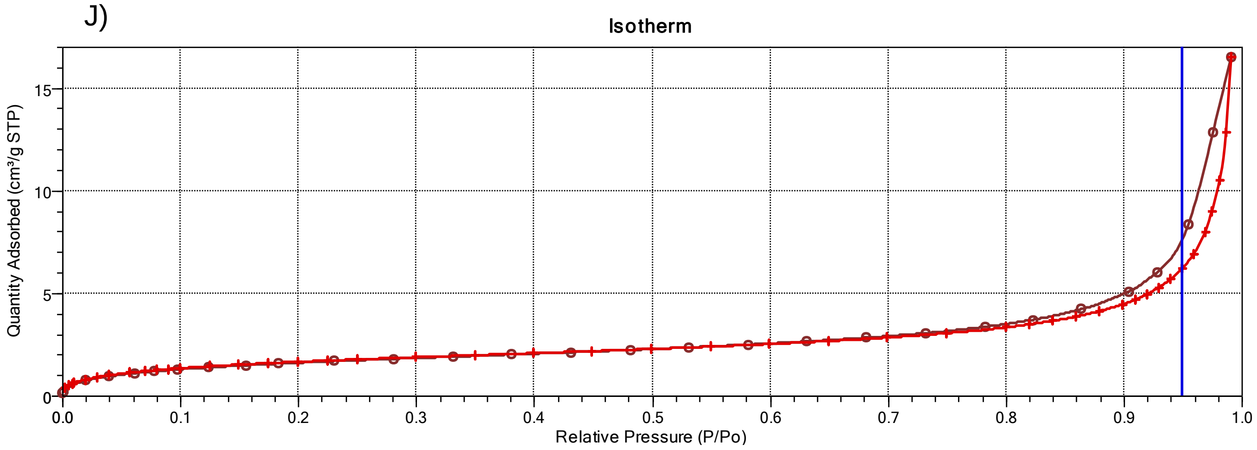

Figure S3: Isotherms of the UFX5-pHt membranes samples fouled with thermo-mechanical pulping process water. A) reference with water, B) second reference with water, C) first rerun of second reference with water, D) second rerun of second reference with water, E) third reference with water, F) first rerun of third reference with water, G) second rerun of third reference with water, H) constant pressure fouling with thermomechanical pulping process water, I) first rerun of constant pressure fouling with thermomechanical pulping process water, J) second rerun of constant pressure fouling with thermomechanical pulping process water.

A)

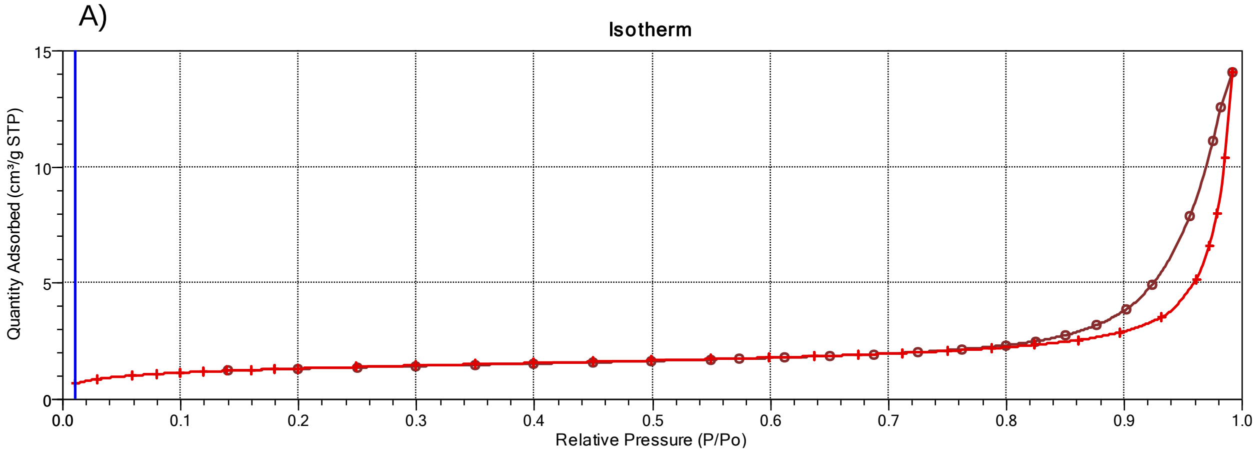
Isotherm
Quantity Adsorbed (cm³/g STP)
Relative Pressure (P/Po)
0
5
10
15
0.0
0.1
0.2
0.3
0.4
0.5
0.6
0.7
0.8
0.9
1.0

B)

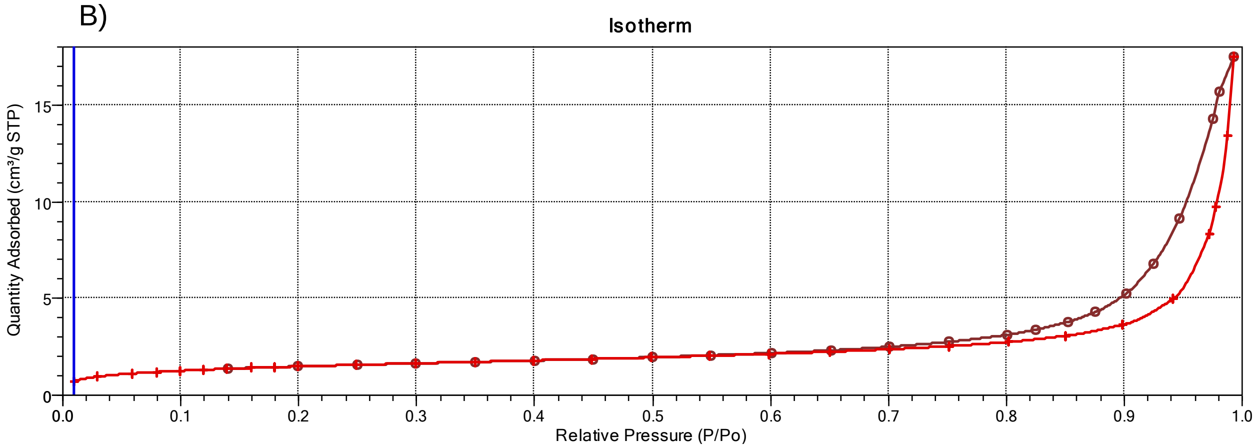
Isotherm
Quantity Adsorbed (cm³/g STP)
Relative Pressure (P/Po)
0
5
10
15
0.0
0.1
0.2
0.3
0.4
0.5
0.6
0.7
0.8
0.9
1.0

C)

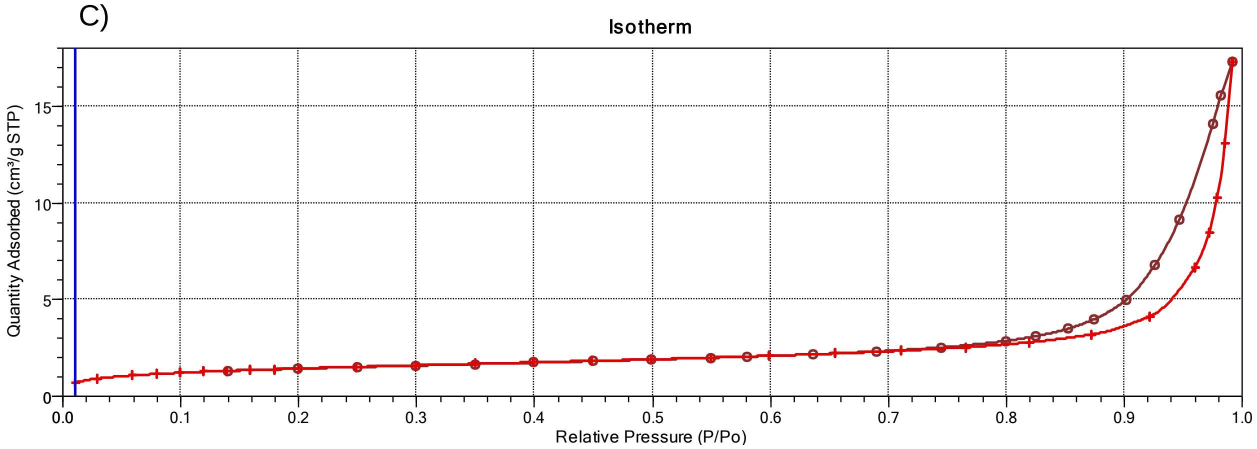
Isotherm
Quantity Adsorbed (cm³/g STP)
Relative Pressure (P/Po)
0
5
10
15
0.0
0.1
0.2
0.3
0.4
0.5
0.6
0.7
0.8
0.9
1.0

D)

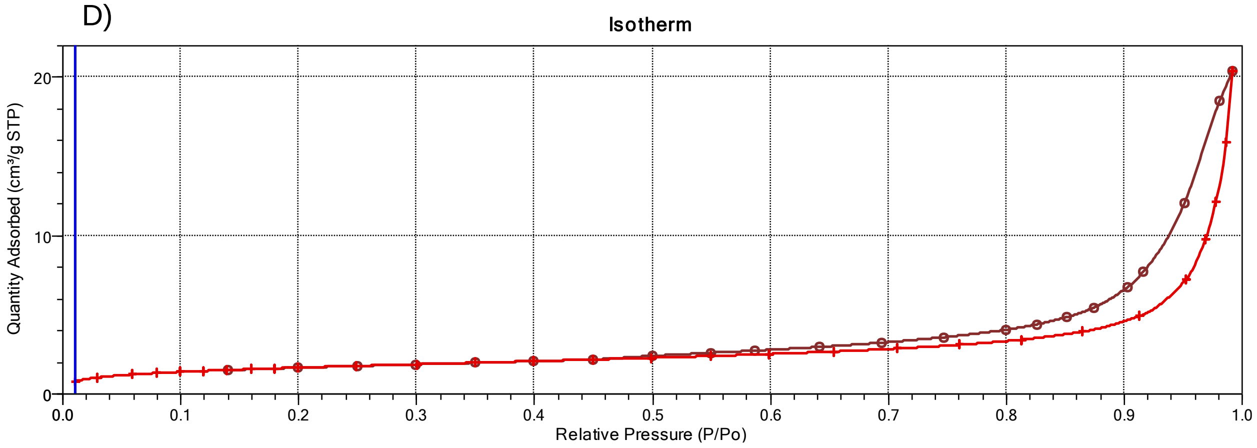

E)

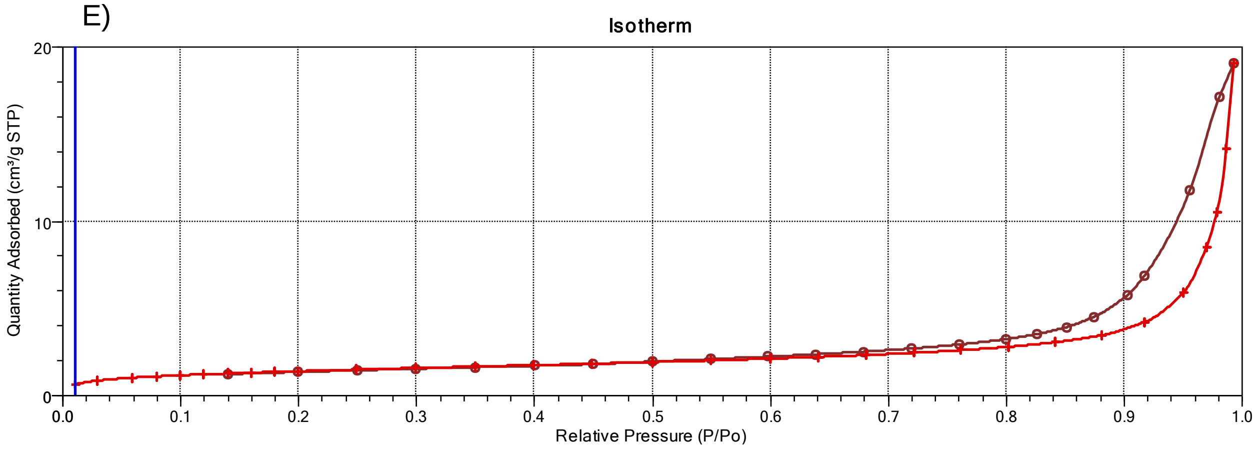

F)

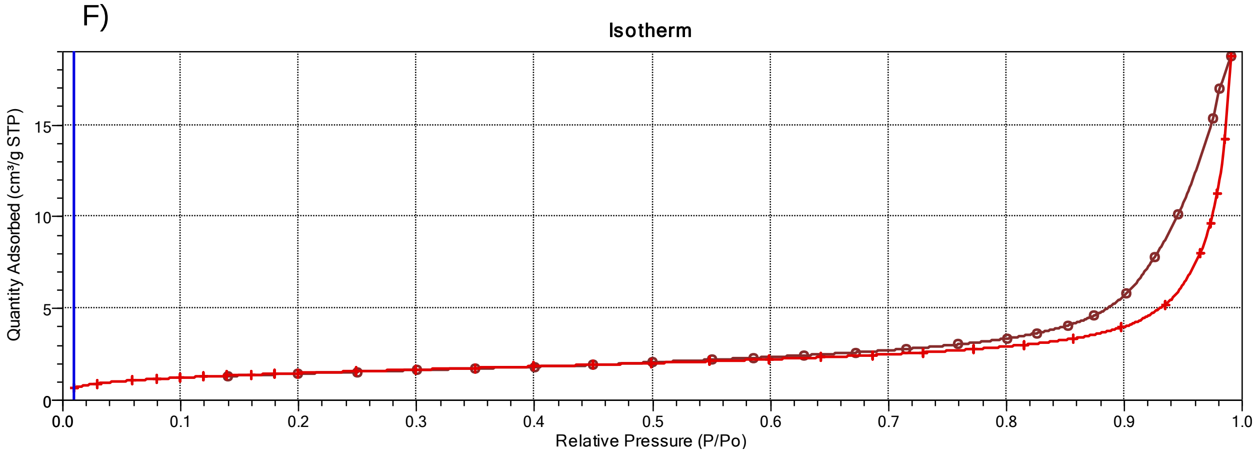

Figure S4: Isotherms of the UP010 membranes samples fouled with pressurized hot-water extract. A) reference with water at 25 °C, B) reference with water at 60 °C, C) adsorptive fouling with spruce extract at 25 °C, D) adsorptive fouling with spruce extract at 60 °C, E) constant pressure fouling with spruce extract at 25 °C and F) constant pressure fouling with spruce extract at 60 °C.

A)

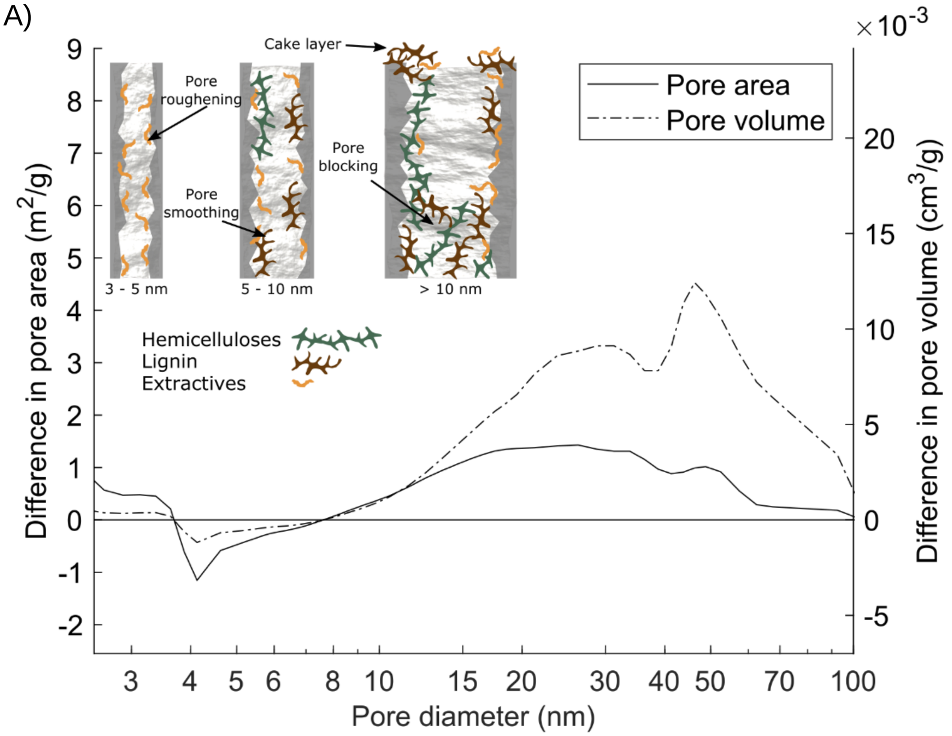

Cake layer
Pore roughening
Pore smoothing
Pore blocking
3 - 5 nm
5 - 10 nm
> 10 nm
Hemicelluloses
Lignin
Extractives
Pore area
Pore volume
Difference in pore area (m²/g)
Difference in pore volume (cm³/g)
×10⁻³
Pore diameter (nm)

B)

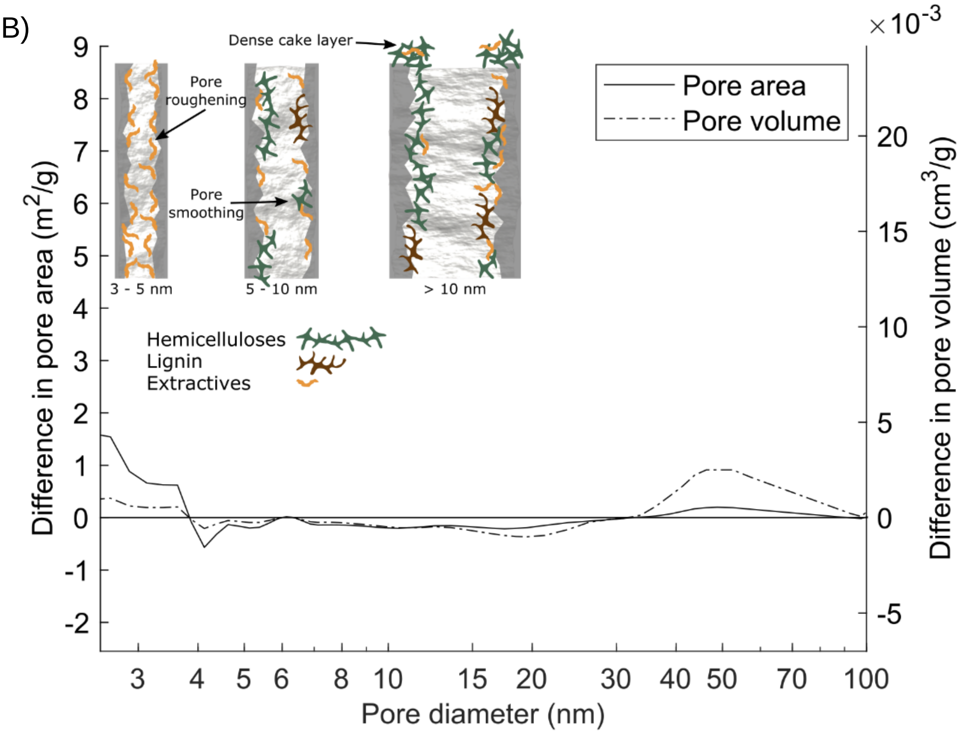

Dense cake layer
Pore roughening
Pore smoothing
3 - 5 nm
5 - 10 nm
> 10 nm
Hemicelluloses
Lignin
Extractives
Pore area
Pore volume
Difference in pore area (m²/g)
Difference in pore volume (cm³/g)
×10⁻³
Pore diameter (nm)

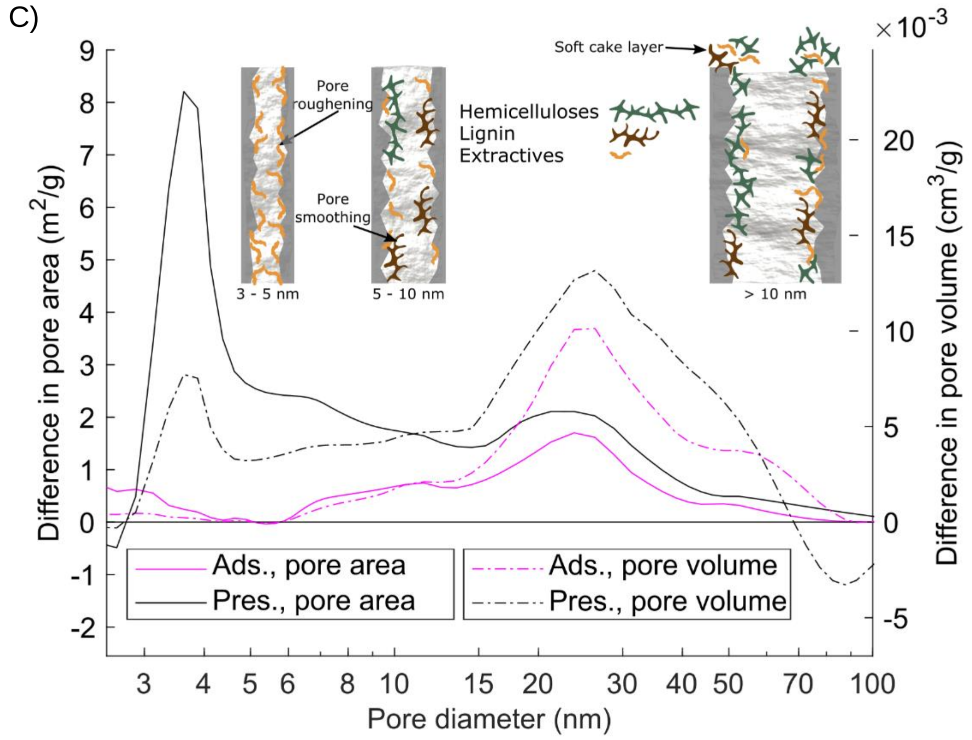

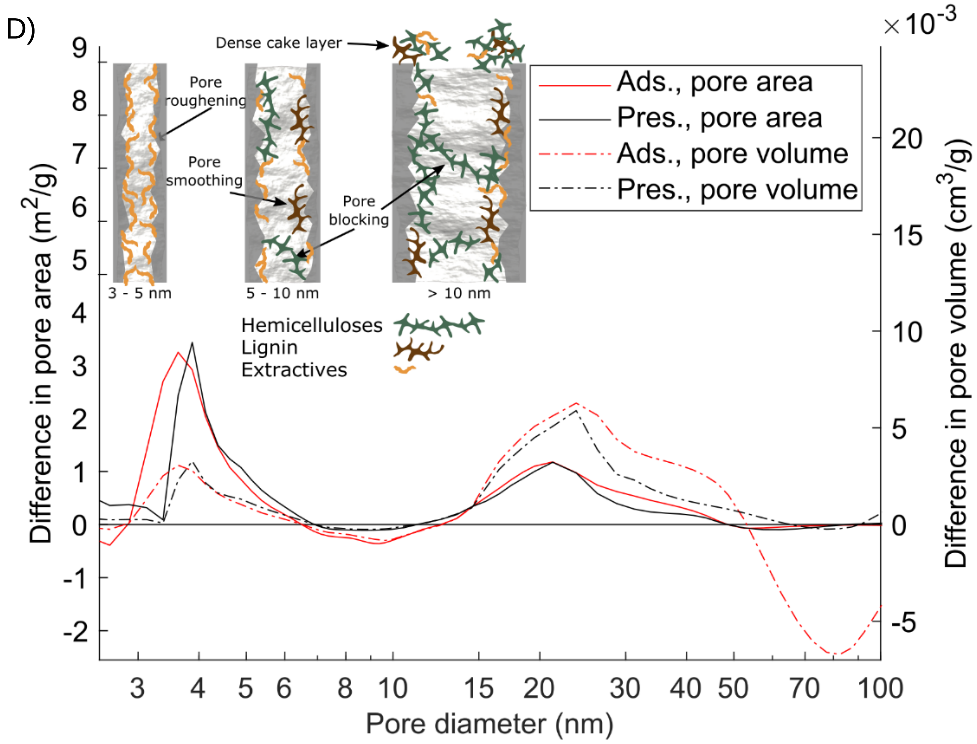

Figure S5: Differences in pore area and pore volume distributions after membrane fouling and illustrations for fouling layers formed by A) black liquor, B) thermomechanical pulping process water, C) pressurized hot-water extract at 25 °C and D) pressurized hot-water extract at 60 °C. Each difference plot was obtained by subtracting the pore area or pore volume distribution of reference membrane from the corresponding distribution of fouled membrane (presented in the manuscript in Figure 1.) [The illustrations for fouling layers were created with Inkscape version 0.92 (https://inkscape.org/).]
